# Improvement of long-term memory access with a pro-dopamine regulator in an elderly male: Are we targeting dopamine tone?

**DOI:** 10.15761/JSIN.1000165

**Published:** 2017-06-17

**Authors:** Thomas McLaughlin, David Han, James Nicholson, Bruce Steinberg, Kenneth Blum, Marcelo Febo, Eric Braverman, Mona Li, Lyle Fried, Rajendra Badgaiyan

**Affiliations:** 1 Center for Psychiatric Medicine, Lawrence, MA, USA; 2 Department of Management Science and Statistics, University of Texas at San Antonio, San Antonio, TX, USA; 3 Mitech Surgical Products, Dedham, Massachusetts, USA; 4 Department of Psychology, Curry College, Milton, MA, USA; 5 Department of Psychiatry & McKnight Brain Institute, University of Florida College of Medicine, Gainesville, FL, USA; 6 Department of Psychiatry and Behavioral Sciences, Keck Medicine University of Southern California, Los Angeles, CA, USA; 7 Division of Applied Clinical Research & Education, Dominion Diagnostics, LLC, North Kingstown, RI, USA; 8 Department of Neurogenetics, Igene, LLC, Austin, TX, USA; 9 Department of Addiction Research & Therapy, Nupathways, Inc. Innsbrook, USA; 10 Department of Clinical Neurology, PATH Foundation NY, New York, USA; 11 Division of Neuroscience Based Addiction Therapy, The Shores Treatment & Recovery Center, Port Saint Lucie, FL, USA; 12 Eötvös Loránd University, Institute of Psychology, Budapest, Hungary; 13 Department of Psychiatry, Wright State University Boonshoft School of Medicine and Dayton VA Medical Center, Dayton, OH, USA (IE); 14 Division of Precision Medicine, Geneus Health LLC, San Antonio, TX, USA

**Keywords:** long-term memory, animal naming test, pro-dopamine regulator (KB2200Z), dopamine tone

## Abstract

With aging, there is decline in both short-term and long-term memory. This effect is magnified by epigenetic insults on specific, dopamine- related genes (e.g., DRD2, DAT1) as well as by impaired or reduced mRNA transcription. In addition, long-term memory ability is positively correlated with dopamine function and there is evidence that aging is associated with a reduction in brain dopamine D2 receptors, with an acceleration seen in aging-induced dementia. As a result, the authors tested the acute effect of a Pro-Dopamine Regulator (KB220Z, liquid Nano variant) on an aspect of long-term memory performance in a 77-year-old, highly functional male, using the Animal Naming Test (ANT). An improvement in long-term memory retrieval had initially been noted during the subject’s follow-up neurology exam, after he had been, for other reasons, taking KB220z. The patient had been given a number of ANTs by his primary and, later, another neurologist, from 2013 to 2016. Because the number of ANT observations was small (N = 7 with two groups) and the data uncorrelated, a non-parametric Wilcoxon-Mann-Whitney test was performed to test mean differences. After KB220z, the patient had much higher scores (p = 0.04762) on the ANT vs. when not taking it. His scores increased from the 30^th^ percentile (pre-test) to the 76^th^ percentile, after the first administration of KB220z and, later, to the 98^th^ percentile, after a second administration of KB220z, six months later. The results indicate that KB220z, given acutely, increased a form of long-term memory retrieval in a highly functional, elderly male. Larger, double-blind, randomized controlled studies are encouraged.

## Introduction

A PubMed search (2-22-17), with the terms, “dopamine and cognition” resulted in 3619 citations. An additional search revealed that a hypo-dopaminergia trait (genetic) or state (epigenetic) predisposes to cognitive decline, especially, with aging. Over 650 studies emphasized this relationship between dopamine and memory [1–19].

Striatal dopamine (DA) functions, affecting cognitive performance, have been linked to the Taq I A polymorphism of the DRD2/ANKK1 gene. In humans, the A1 allele of the DRD2/ANKK1-TaqIA polymorphism is associated with reduced density of striatal DA D2 receptors [20].

In addition, Persson, Riekmann, Kalpouzos, *et al.* [21] have demonstrated that older A1-carriers exhibit worse memory performance during long-term memory updating, compared to A2 non-carriers. The exacerbation of the effects of low dopamine upon on memory and cognition, with aging, has been extensively demonstrated [22–24].

### Rationale for case report utilizing KB220z

Genetic antecedents and, in particular, polymorphisms, have been linked to a hypo-dopaminergic trait, resulting in an impairment in global cognition [25–28]. The clinical benefits of KB220 variants in modifying this trait, have, over a fifty-year period been documented by Blum, *et al.* [25].

Based on the notion that hypo-dopaminergia may be a root cause of dementia, the authors tested the acute effects of a Pro-Dopamine Regulator (KB220Z, liquid Nano variant) on long-term memory performance in a 77-year- old, highly functional male, using a test of semantic verbal fluency, known as, the “Animal Naming Test” (Mesulam [29]). Additional reasons for using KB220, in this context, included the possibility that the low dopamine tone, seen in the elderly, might also be responsible reduced focusing ability. In fact, Badgaiyan *et al.* [30] study of the resting tone of dopamine in ADHD supports this notion, since tonic release of dopamine was diminished but its phasic release enhanced in the right caudate of these patients.

Combining LORETTA research on ADHD [31] as well as with the reported dopamine links [32,33] between the EEG index, P300, and memory [34–39] may offer ways of measuring the role of KB220Z in promoting dopamine homeostasis.

### Animal naming test-a test of semantic flexibility retrieval

Mesulam’s, Principles of Behavioral and Cognitive Neurology [29] describes cognitive tests used to assess memory. The generation of lists of words, beginning with a specified letter (*i.e.*, lexical) or which are categorically related (*i.e.*, semantic) can be used to assess the mental functions of perseverance, fluency and mental retrieval. On tests of semantic list generation (animals), in one minute, older adults produce an average of 18 (+/− 5), depending upon age and education. Successful performance on these tests implies normal perseverance and scanning of internal, long-term memory registers.

Patients unable to sustain effective, behavioral output during this test may also show generalized slowing and delayed response times. These characteristics are also important features of abulia- a hypo-dopaminergic state seen following frontal lobe lesions. In particular, Choutier, *et al.* [40] found that, especially, in abulic states, semantic fluency performance is related to left middle temporal lesions.

In addition, the accessing of “long-term memory” stores, in the Animal Naming Test, involves accessing “semantic memories” (or general knowledge). This activity requires the operation of organized neural networks, connecting sub-cortical and cortical areas in order to access and decode long-term memory stores. The ability to retrieve correctly (without breaking the rules) a limited set of words demands planning, monitoring, judgment, and decision-making as well as the ability to inhibit irrelevant information and select the correct responses (frontal lobe functioning), (Oria, *et al.* [41]).

With respect to the selection and related inhibition of attempts at accessing information stores, Stelzel, *et al.* [42] note that genetic and pharmacological studies indicate an important role of the dopamine D2 receptor (DRD2) in flexible, behavioral adaptation, mostly, with respect to reward-based, learning paradigms [43,44]. In addition, evidence from imaging genetics also indicates that intentional, cognitive flexibility, which is mediated by the lateral frontal cortex, is affected by variations in DRD2 signaling.

### Hypothesis

Based on these studies, the authors hypothesized that the use of KB220z (as a liquid Nano) would increase long-term memory ability, as measured by Animal Naming Test performance in a highly functional, elderly male, through its impact on dopamine regulation/balance.

## Methods

The study has been approved by the PATH Foundation NY IRB Committee as part of an approval provided for the testing of the Pro-dopamine regulator (KB220Z). The actual study was performed by Thomas McLaughlin, MD, PhD at the Center for Psychiatric Medicine. The subject of the case study, AC, provided written informed consent for his medical information to be used in the case report. Statistics was performed by David Han, PhD at the University of Texas, San Antonio, Texas.

The most recent variant of KB220Z (liquid Nano) is comprised of the following ingredients:

Thiamine, 15 mg (1033% of Daily Value), Vitamin B6, 10 mg (500%), Chromium poly nicotinate 200 mcg (166%), and a fixed dose combination of amino acids and herbs, called Synaptose.

Synaptose contains DL-Phenylalanine, L-Tyrosine, Passion Flower Extract, a complex containing Arabinogalactans, N-Acetylglucosamine, Astragalus, Aloe Vera, Frankincense Resin, White Pine Bark Extract, and Spirulina, Rhodiola, L-Glutamine, 5-Hydroxytryptophan (5-HTP), Thiamine Hydrochloride, Pyroxidal-5-phosphate and Pyridoxine HCl.

### Case presentation and results

The patient, AC, was a 73-year-old, highly educated male, with a Master’s degree in Engineering. He was first seen by his primary care physician for an evaluation, for having fallen asleep twice, while driving, in February, 2013. To rule out underlying diagnostic possibilities, such as, syncope, seizures, narcolepsy and excessive daytime sleepiness, he was referred for a neurological evaluation in March, 2013. (The routine ordering of a complete battery of neuropsychological tests in the US is not readily reimbursed by insurance companies as being “medically necessary”).

Screening, neurobehavioral batteries, such as, those employed by the treating neurologist, included inter alia, the Mini-Mental Status Test, the Clock Face Drawing Test, the immediate and delayed recall of words/phrases after 3–5 minutes, with the interposition of distracting testing of language (confrontational naming of common objects, repetition of phrases (“No ifs, ands or buts”), tests of apraxia, naming and sequencing American presidents, spelling the word, “WORLD,” backwards, etc. (These neurobehavioral tests are also used to measure the therapeutic effects of prescribed medications, such as, galantamine),

The presence of “neglect,” a neurological symptom resulting from right parietal lobe damage, can be assessed by the patient’s performance on the Clock Drawing Test. The patient must first “draw a clock face, with evidence for “neglect” seen in a failure to include the numbers on the left side of the clock face. In addition, a perceptual, spatial orientation defect can also be detected with this test by the incorrect placement of the hands of the clock at the prescribed time, e.g., “10 minutes after 11:00 o’clock”.

In addition, the ability to remember only one of three phrases after 3–5 minutes is considered “moderate short-term memory impairment,” with the ability to remember two of three, constituting “mild short-term memory impairment”.

### History of present illness

The patient reported to the neurologist that he had driven 120 miles in one hour and 50 minutes, on February 21, 2013. He was tired, had skipped breakfast and lunch, had only drunk a bottle of water and had had no food that day. He had missed his usual 2 cups of morning coffee. He had a cough. Twice, while driving, he awoke from sleep, while drifting out of his lane and heading towards the highway divider.

The following day, he had worsening cough, productive of green sputum. Chest x-ray demonstrated bilateral infiltrates. He was prescribed Levaquin 500 mg daily for 7 days for pneumonia and reported a rapid recovery.

(He reported no history of sleep paralysis, hallucinations, cataplexy, or sleep attacks). To exclude other possible causes of reasons for his near loss of consciousness, the patient underwent an EEG the next day. This recording, carried out in a sleep laboratory, under both awake and sleep conditions, revealed no epileptiform activity, signs of narcolepsy, or sleep apnea.

### Review of systems

The patient reported an ongoing problem with sleep, attributed to an uncomfortable mattress. There was a tendency to snore as well as a tendency to fall asleep, while watching TV. He had never before fallen asleep, while driving.

### Physical exam

On exam, the patient’s head was normo-cephalic and atraumatic. Sclera was white and pupils equal and briskly reactive to light. Nose and throat were unremarkable. There full range of motion of neck, without meningismus. Carotid arteries were without bruit. Lungs were clear. Heart sounds were regular, with a holo-systolic murmur.

On neurological examination, AC had a completely normal mental status. He was alert, oriented and attentive. He had normal speech without dysarthria or aphasia. He had no apraxia and no neglect. He had normal memory for events and fund of knowledge.

On the cranial nerve exam, pupils were equal in size and briskly reactive to light bilaterally. Funduscopic exam revealed sharp optic disc margins, without disc pallor. He had full visual fields and ocular motility, without nystagmus. He had normal facial sensation and a full smile. Hearing was intact to conversational speech. Palate elevated well. Sternocleidomastoid and trapezius strength were full and tongue protruded in the midline.

On the motor exam, he had no involuntary movements. There was no pronator drift. Strength was 5/5 in both upper and both lower extremities. He had a normal cerebellar examination, with normal coordination on finger-to-nose, heel-to-shin testing as well as rapid alternating movements of the upper extremities. He had normal stance and gait. He was able to take steps on heels, toes and perform tandem gait. Sensory exam was intact to pinprick and vibration was present at feet. Deep tendon reflexes were 2 + throughout and toes were bilaterally down-going, on Babinski testing.

### Assessment

#### Mental status change

This, now, 73-year-old man reported falling asleep twice, while driving on February 21, 2013. There were extenuating circumstances, *viz.*, he had skipped breakfast and lunch that day and had only drunk a bottle of water. He had missed his usual 2 cups of coffee. He had reported a cough, productive of green sputum. The following day, he was diagnosed with a respiratory infection, with bilateral infiltrates on chest x-ray.

To evaluate the risk of recurrent episodes of falling asleep, while driving, the patient was evaluated in the hospital sleep laboratory for sleep apnea, with an overnight sleep study. This workup was negative for sleep apnea or other sleep disorders.

Routine laboratory studies were normal. Vitamin B-12, Vitamin D3 levels, Folate and an Iron Profile were normal. EKG results indicated a Left Bundle Branch Block.

#### Neurological visit

On 3-11-2015, AC returned to the office for memory concerns, at the behest of his wife, although he denied any significant memory impairment.

On neurological examination, he was alert, attentive and cooperative. There was no fluctuation of alertness or lucidity, which findings ruled out delirium. He incorrectly thought the day of the week was “Wednesday” rather than “Tuesday”. He initially provided the date as the “15tht” rather than the “11th,” however, was able to correct himself.

He did not know the county the hospital was in. He registered 3 out of 3 objects at 0 minutes (immediate memory) and recalled 1 out of 3 objects at 5 minutes, without cues, and 2 of 3 objects, with cues (short-term memory). He made one error on serial 7s testing and correctly spelled the word, “WORLD,” backward. He did not copy the intersecting pentagons accurately. He scored 25/30 on the Mini-Mental Status exam. He correctly drew a clock face and provided the correct time. He generated a list of 15 animals over 60 seconds on the Animal Naming Test.

He had normal speech, without dysarthria or aphasia. He named all 6 items on the Stroke Card and described the Cookie Jar Picture in detail. He had no apraxia and no neglect.

On cranial nerve exam, pupils were equal in size and briskly reactive to light bilaterally. Funduscopic exam revealed sharp optic disc margins without disc pallor. He had full visual fields and ocular motility, without nystagmus. He had normal facial sensation. He had a full smile. Hearing was intact to conversational speech. Palate elevated well. Sternocleidomastoid and trapezius strength were full and tongue protruded midline. On motor exam, he had no involuntary movements. There was no pronator drift. Strength was 5/5 in both upper and lower extremities. He had normal coordination on finger-to- nose testing. He had normal stance and gait. He was able to perform tandem gait. Sensory exam was intact to light touch.

#### Memory lapses or loss

AC had evidence of moderate memory loss. He was started on donepezil 5 mg, 1 tablet daily in the evening.

#### Neuro-radiological testing

On 3-16-15, the patient underwent a MRI brain scan, without contrast. T1-weighted axial, sagittal, coronal, T2-weighted coronal, gradient echo, FLAIR, diffusion-weighted, ADC map axial images were obtained. The study was performed on a 1.5 Tesla magnet.

### Indication

#### Memory changes

##### MRI Findings

“No extra-axial collection is seen. Possibly calcified/ossified dural plaques are overlying both frontal convexities. The ventricular system and extra-axial CSF-containing spaces are plump, suggestive of mild global atrophy. There are no territorial infarcts, acute bleeds or large masses. A few, scattered, small, supra-tentorial, white matter T2 hyper-intensities are non-specific in appearance. These can be related to mild small vessel disease. No obvious mass effect is seen. No areas of diffusion restriction are identified to suggest acute infarcts. Normal flow-voids are present. The cranio-cervical junction is unremarkable. The impression is that there is no acute infarct, hemorrhage, discrete mass but there was is mild global atrophy”.

#### Neurological visit

On 3-16-15, AC returned for a neurological follow-up, with his wife present, following his brain MRI test. The patient had begun taking donepezil 5 mg nightly. He had tolerated this medication well and had experienced 4–5 headaches during the month.

On neurological exam, the patient was alert, attentive and cooperative. He did not know the correct day of the week nor the month. He was oriented to the year. He did not know the county the hospital was in. He registered 3 out of 3 objects at 0 minutes and recalled 1 of 3 objects at 5 minutes, without cues, and 2 of 3 three objects, with cues. He made one error on serial 7s. He spelled “WORLD” correctly, backward. He did not copy the intersecting pentagons accurately.

Overall, he scored 25/30 on the Mini-Mental Status exam. He was able to complete a clock face and provide the correct time. He generated a list of 15 animals within 60 seconds on the Animal Naming Test. He had normal speech without dysarthria or aphasia. There was no evidence of apraxia or neglect.

In summary, AC evidenced moderate, short-term memory loss. He was tolerating donepezil 5 mg nightly and his dose was to be increased to 10 mg nightly, after a month, to avoid side effects from too rapid titration.

His brain MRI was reviewed. It indicated limited, white matter changes; most likely the result of small vessel disease as well as atrophy, possibly, in excess of what one might anticipate for his age. The atrophy was non-specific in nature.

#### Neurological visit

On 4-2-15, AC was seen in neurological follow up. He had been hospitalized, because of worsening confusion for one week (had begun donepezil 2 weeks earlier), reduced bladder control, abdominal discomfort and heme-positive stool. Donepezil was discontinued.

On neurological examination, AC was found to be alert and oriented X 3. He registered 3 words at 0 minutes and recalled 1/3 words after 5 minutes. He was able to spell “WORLD” backward and perform serial 7s, with one error.

Speech was fluent with no paraphasic errors. He had normal comprehension, repetition, naming, reading and writing. No neglect or apraxia was noted. He was able to copy intersecting pentagons and place the numbers on a clock, although the hands were misplaced. Mini-Mental Status Test score was 27/30. He generated a list of 12 animals in 60 seconds on the Animal Naming Test.

In summary, the patient was thought to have had an adverse response to donepezil, with increased confusion. Donepezil was discontinued. He and his wife were instructed to monitor his status for the next two weeks. Galantamine was begun, initially, at an 8 mg dose, with a further increase to 16 mg.

#### Neurological visit

On 9/25/2015, AC returned for follow-up neurological examination.

He was alert and oriented X 3. He registered 3 words at 0 minutes and recalled 1/3 words after 5 minutes. He spelled “WORLD” backward and performed serial sevens, without error. Speech was fluent with no paraphasic errors.

The patient had normal comprehension, repetition, naming, reading and writing. No neglect or apraxia was noted. He was able to copy intersecting pentagons and place the numbers on a clock, with clock hands in good position. He scored 27/30 on the Mini-Mental Status Test.

The patient generated a list of 15 animals in 60 seconds on the Animal Naming Test. His short-term memory had subjectively improved on galantamine 16 mg.

#### Neurological visit

On 3-28-2016, AC, now, 77- years old, was seen for his original finding of moderate memory loss. He had been stable, since last observed.

Prior to this visit, the patient, at the behest of the first author (TM), in order to enhance AC’s concentration ability (about which he had complained), had been taking liquid Nano, KB220zZ variant regularly.

After self-administration of this nutraceutical for a few days, he felt best, when taking ¾ oz. in the AM. Within two days of taking this putative, Pro-dopamine regulator, he reported he felt “mentally much sharper” and was suddenly able to “multi-task.” He noted that his tendency to procrastinate was markedly improved. (AC had been asked by a nationally famous author, whose book discussion he had attended, whether he could provide this author with a copy of AC’s Master’s thesis. The subject matter of the thesis was related to the author’s latest book. Two months, later, but one day after starting KB200Z, AC found the thesis in his former university’s library, had it copied, and mailed it to the author).

Two and a half hours prior to his neurological evaluation of 3-28-2016, the patient had taken ¾ oz of KB220Z, without food. On examination, AC was alert and oriented X 3. He was able to register 3 words at 0 minutes and recall 1/3 words after 5 minutes. He was able to spell “WORLD” backward correctly and perform serial 7s with 1 error. His speech was fluent with no paraphasic errors. He had normal comprehension, repetition, naming, reading and writing. There was no evidence of neglect or apraxia. He copied intersecting pentagons, placed numbers on a clock, and set the clock hands correctly.

**Figure 1:**
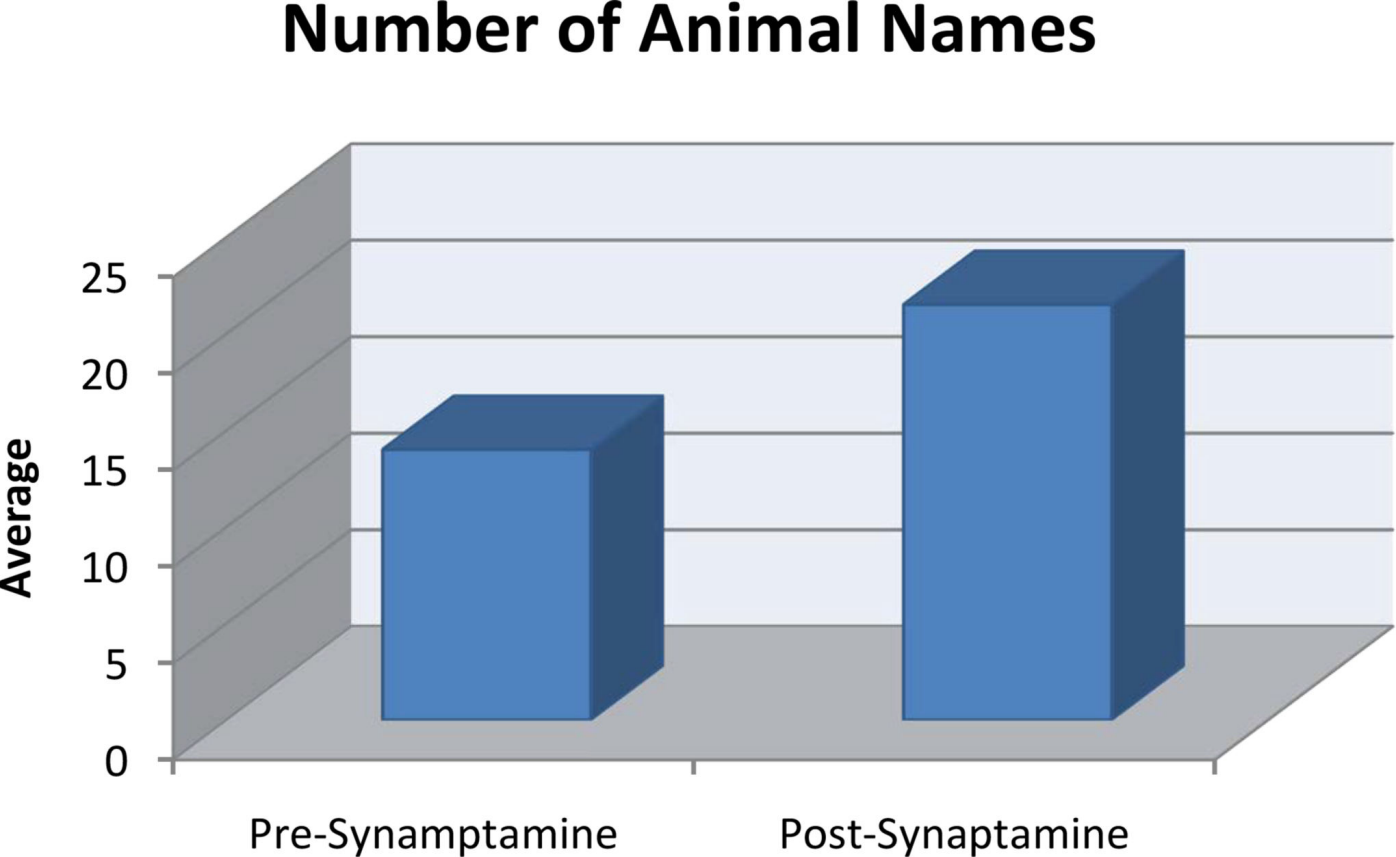
Mean animal naming scores for the pre-synaptamine and post-synaptamine conditions Displays a statistically significant difference between pre-and post- KB220z (in the form of Synaptamine) with a higher score post- KB200z (*p*-value = 0.04762).

AC scored higher on the Mini-Mental Status test, 28/30. He now generated 19 animals in 60 seconds on the Animal Naming Test (Figure 1). On his current regimen, KB220Z made him feel “mentally sharper,” beyond anything he had previously experienced.

With this improved outcome on the Animal Naming Test, it was decided (by TM and KB) to re-evaluate AC to determine if the KB220Z variant could produce an enhanced, positive Semantic Verbal Fluency Test/Animal Naming Test result.

### Experimental neurological examination after a 10-day washout of KB2200z

On 10-17-16, following a ten-day washout, during which AC stopped KB220z, neurobehavioral testing was carried out. During testing, he was incorrectly oriented to date and day but correctly identified month and season. He was unable to name the current President but did so with cues. He was unable to name the current President’s predecessor but also did so with cues. He correctly spelled the word, “WORLD,” backwards. Language repetition was normal but he incorrectly named the face of a watch as “the window.” Reading comprehension and writing were normal. There was no left/right confusion nor was there any apraxia. He correctly entered the numbers on a clock face and set the time as requested. There was no neglect. Registration of words and phrases at zero minutes was normal. He remembered 0 of 3 words/phrases after 3 minutes and recalled 1 word with cues. He correctly named the direction of travel from New York City to Arizona as “West”. Digits forward was 7 and Digits Backward was 4. AC generated 13 animals in one minute on the Animal Naming Test (Figure 1).

### Experimental neurological examination two hours after taking KB220Z

**Figure 2:**
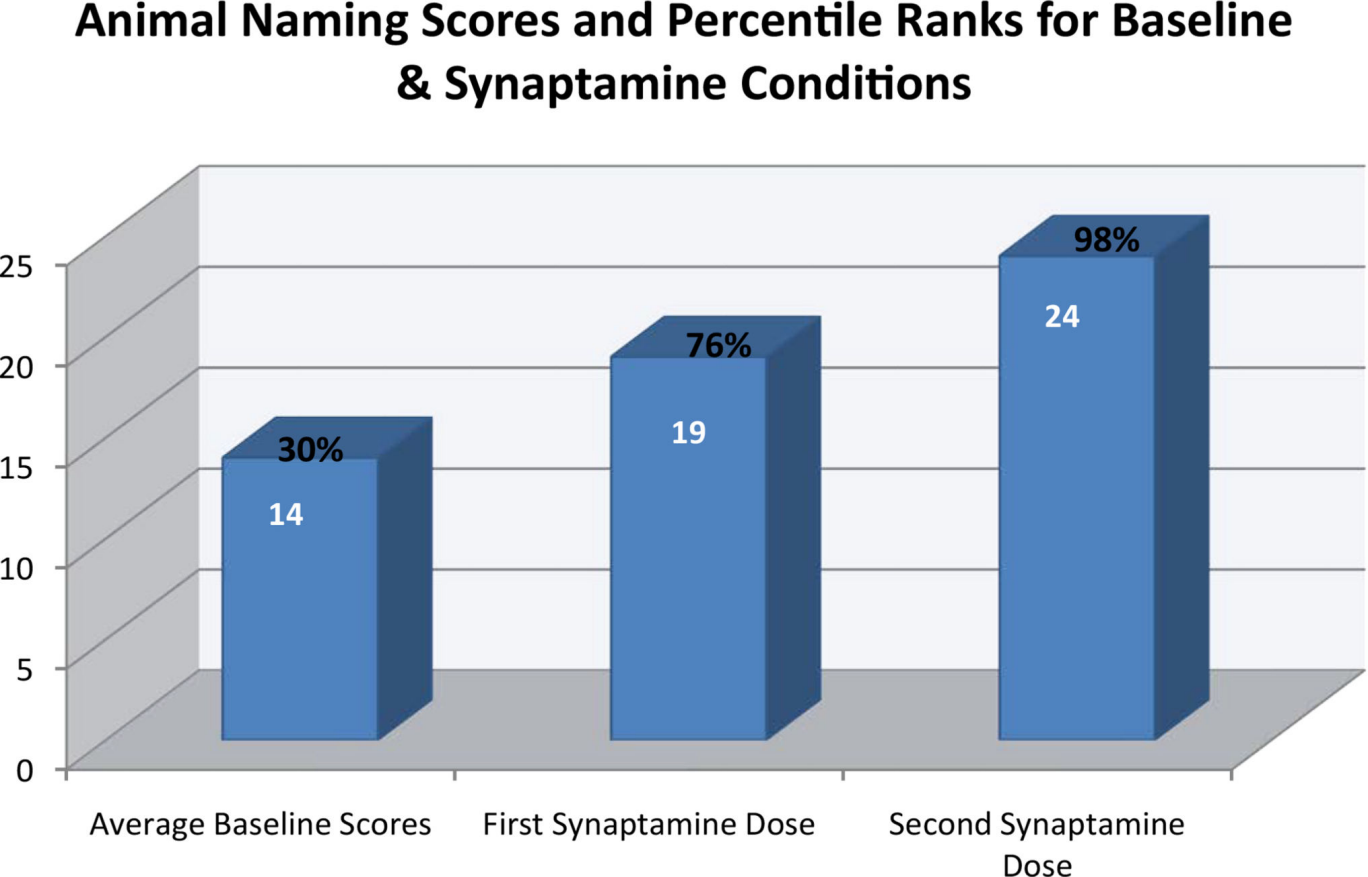
Raw animal naming test scores and their percentile ranks for the average of the baseline conditions and two KB220z administrations Percentile ranks for the raw, animal naming scores were derived from the normative data for the Semantic Verbal Fluency Test [45]. The authors reported a mean score of 16.1 and a standard deviation of 4, for a sample of 228 individuals in the age range of 70–79 years. Our data reveal that, on average, before the administration of Synaptamine, AC’s performance on the Animal Naming Test was in the 30^th^ percentile. The first administration of Synaptamine improved his performance (76^th^ percentile). A second administration of Synaptamine, six months later, elevated his score to the 98^th^ percentile.

On 10-18-2016, AC took ¾ oz of KB220Z in the liquid nano form, without food, two hours prior to a neurological examination. He was incorrectly oriented to date and day but correctly identified the month and season. He correctly named the President, but in naming his predecessors, reversed the order of GW Bush and Bill Clinton but corrected these with cues. He was unable to name Clinton’s predecessor but did so with cues. He incorrectly spelled the word “WORLD”, backward as “WOLD”. Language repetition was normal but he again incorrectly named the face of a watch as “the window”. Reading comprehension and writing were normal. There was neither left/right confusion nor apraxia. He correctly entered the numbers on a clock and set the time correctly. Registration of words and phrases at 0 minutes was normal. He remembered 0 of 3 words/phrases after 3 minutes and recalled 1 word, with cues. (Thus, short-term memory did not improve to any clinically relevant extent). He correctly named the direction of travel from New York City to Arizona as “West”. His Digits Forward was 7 and Digits Backward was 4. On KB220Z, the patient generated 24 named animals in 1 minute (Figures 1 and 2, and Table 1).

### Statistical analysis

Since the sample size is small and the data uncorrelated (n = 7, with two groups), the non-parametric, permutation-based, Wilcoxon-Mann-Whitney test (an alternative to the t-test) was performed. Instead of sample size, multiple observations served as the sample. Test data collected here are not traditional or independent or paired samples for running parametric or non-parametric tests. However, after serious consideration, a simple Wilcoxon Test was chosen, to be conservative about test results. The test was statistically significant, indicating that the patient, after taking KB2200Z, had a higher Animal Naming Test score (p-value = 0.04762) (Figure 1).

The Figure 2 provides greater detail related to the effect of KB220z on AC’s retrieval from long-term memory stores. The information related to improvement by rank is a relevant factor in explaining the findings.

## Discussion

A highly educated, elderly male, with moderate memory impairment was originally given KB200Z to aid his focusing ability. During follow-up, neurological testing for a short-term, memory problem, AC’s capacity to “name as many animals as possible in 60 seconds” was routinely evaluated. There was no appreciable improvement in his short-term memory, with trials of donepezil and galantamine. Donepezil was discontinued due to side effects and galantamine had been routinely taken on an empty stomach, contrary to instructions.

When the patient told one of the authors (TM) that he had done extremely well on the Animal Naming Test, during the last visit to his neurologist and that his performance improvement was likely due to his having taken KB200z, the team decided to review his overall, neurobehavioral test performance over his five follow-up neurological visits.

Off KB200z, scores on the Animal Naming Test, during four examinations were: 15, 15, 12, and 15.

*On* ¾ oz KB200z, taken 2 hours before his follow-up visit with his treating neurologist, his Animal Naming Test score was 19.

Following the assessments by his treating neurologist, an independent, board-certified neurologist conducted two, analogous, neurobehavioral examinations; *On* and *Off* KB200z.

*Off KB200z*, AC’s Animal Naming Test score was 13. The next day, following an overall wash-out period of 10 days, AC took ¾ 0z of KB200z. on an empty stomach. Two hours later, he was given a neurobehavioral examination, which included the Animal Naming Test and achieved a score of 24. The results represented a marked difference between the *ON* and *OFF* KB200z conditions.

Inspection of his performance on other tests included in the neurobehavioral examinations indicated a *selective effect* on Semantic Verbal Fluency (Animal Naming Test), with no noticeable effect on tests of short-term memory, language, spatial orientation, etc.

It is noteworthy that Rondanelli *et al*. [46] found that diet integration of the precursor amino acid tryptophan, administered to elderly patients, suffering from mild cognitive impairment, after a 12-week period, produced a trend for the semantic verbal fluency improvement (p < 0.06). This finding may be of interest, because one of the ingredients in KB220Z is the downstream precursor to serotonin, 5-hydroxytryptophan (5-HTP). However, the KB220z complex provides other ingredients that activate dopamine reward sites as well [47].

Moreover, in agreement with our hypo-dopaminergic hypothesis [30] Kulisevsky, *et al.* [48] found a significant improvement in the Semantic Verbal Fluency score in a two-year, chronic dopamine replacement follow-up study, using L-Dopa. It is also well known that trans-cutaneous electrical nerve stimulation (TENS) has stimulatory effects on brain dopamine reward sites [49]. Along these lines, Scherder, *et al*. [50] reported that TENS in elderly people improved visual short-term and verbal long-term (recognition) memory as well as semantic verbal fluency. The progressive improvement on the Animal Naming Test may potentially reside in the concept of neuroplasticity as previously suggested in our earlier paper [51] concerning prolonged alleviation of terrifying lucid dreams. The persistent amelioration of these dreams continued for up to 12 months, after a self-initiated, cessation of use of KB220Z. These particular cases raise the scientific possibility that KB200Z increases both dopamine stability as well as functional connectivity between networks of brain reward circuitry in both rodents and humans. The increase in connectivity volume in rodents suggest the induction of neuroplasticity changes, which may be analogous to those involved in human lucid dreaming as well as Rapid Eye Movement sleep. In spite of AC also taking Galantamine which could have impact onto the effects reported here with KB220z, recent evidence suggest that this compound had no effect on two cognitive neurobehavioral measures compared to placebo [52] This finding suggests a possible specific effect of the KB220z.

### Limitations

A single case, observational study represents limited, scientific evidence. Further studies, involving age-matched, otherwise, and neurologically normal subjects and, including placebo, double-blind controls, are needed to establish the validity of these initial but theoretically consistent findings.

## Conclusion

Despite the findings in a single case, the authors note that KB220z was acutely associated with increased access to long-term memory information stores by a highly functional, 77-year-old male. Other researchers are encouraged to also perform larger studies with the aim of attempting to enhance cognition/memory functions in the elderly.

## Figures and Tables

**Table 1:** Statistical analysis of AC’s Animal Naming scores, pre and post-Synaptamine.

Level	Sample Size	Average	Standard Deviation	Standard Error	95% Confidence Interval
**Pre**	5	14.0	1.41	0.6325	(12.24, 15.76)
**Post**	2	21.5	3.54	2.500	(−10.27, 53.27)
